# Case Report: Multidisciplinary management and nursing care for a preterm infant with rare extrarenal malignant rhabdoid tumor: a clinical case study

**DOI:** 10.3389/fped.2025.1638115

**Published:** 2025-09-04

**Authors:** Qiaojia Zhou, Yuanhong Lü, Mingna Chen, Lu Zhang, Qiong Chen, Yuan Li, Queyun Zhou, Xi Huang

**Affiliations:** ^1^ Department of Neonatology Nursing, Shenzhen Children's Hospital, Shenzhen, Guangdong, China; ^2^ Department of Neonatology Nursing, West China Second University Hospital, Sichuan University, Chengdu, China; ^3^ Key Laboratory of Birth Defects and Related Diseases of Women and Children, Sichuan University, Ministry of Education, Chengdu, China

**Keywords:** extrarenal malignant rhabdoid tumor, preterm infant, perioperative nursing, fungal infection, humanistic care, home visits, posttraumatic stress disorder

## Abstract

**Objective:**

To report the multidisciplinary management and perioperative nursing strategies for a preterm infant with extrarenal malignant rhabdoid tumor complicated by fungal infection of the tumor, and to evaluate the effectiveness of humanistic care measures for the family facing a fatal outcome due to malignancy.

**Methods:**

This paper reports the perioperative management and nursing care of a preterm infant with extrarenal malignant rhabdoid tumor using a case study approach. Preoperatively, the management included aggressive anti-infective therapy and isolation measures were applied to prevent fungal transmission, while a multidisciplinary team collaborated to develop a personalized skin management plan to preserve tumor integrity. Postoperatively, comprehensive care measures were implemented, including prevention of perioperative hypothermia, control of wound infection, and refined pain management. During hospitalization, attention was given to the psychological needs of the patient's parents, providing humanistic care and early identification to reduce the risk of maternal post-traumatic stress disorder (PTSD). After discharge, individualized guidance and follow-up home visits were provided to ensure continuity of care, with the medical team, nurses, and family working together to extend the infant' s survival and improve quality of life.

**Results:**

The infant's preoperative condition was effectively managed; the fungal infection was controlled with no nosocomial transmission, and tumor integrity was preserved without new ulcerations. Postoperatively, the infant's physiological functions improved significantly, with no instances of hypothermia or wound infection. Pain scores were successfully maintained at mild-to-moderate levels. The humanistic care approach was associated with positive psychosocial outcomes; the mother's anxiety score decreased from 31 to 17 by discharge, and the parents demonstrated calm acceptance of the infant's prognosis. The infant showed good growth, reaching 4.9 kg at two months of age. Ultimately, the infant survived for 214 days, passing away on February 17, 2024, from tumor recurrence with intracranial metastasis.

**Conclusion:**

A comprehensive, multidisciplinary management approach is critical for rare and aggressive neonatal tumors. In this case, targeted perioperative nursing strategies played a vital role in controlling infection, preserving tumor integrity, and stabilizing the infant's physiological functions. Furthermore, integrated humanistic care was essential in alleviating family psychological stress and improving the infant's quality of life. This case provides a valuable reference for the integrated medical and nursing care of similar challenging cases.

## 1 Introduction

Malignant rhabdoid tumor (MRT) is a rare and highly aggressive pediatric malignancy primarily affecting infants and young children, with an incidence rate of approximately 0.6 per million children (1, 2). This tumor can occur in various sites, including malignant rhabdoid tumor of the kidney (MRTK), atypical teratoid rhabdoid tumor (ATRT) of the central nervous system, and extrarenal extracranial rhabdoid tumor (EERT) (3, 4). In 1978, Beckwith and Palmer (5) first established MRT as a distinct pathological entity from Wilms tumor. Although MRT was initially considered a special subtype of renal Wilms tumor, it not only occurs in the kidney but can also be found in extrarenal organs such as soft tissues, brain, liver, and others. Electron microscopy reveals that MRT lacks the ultrastructural features of rhabdomyoblasts and does not exhibit the typical histological characteristics of Wilms tumor. Consequently, MRT occurring outside the kidney is classified as extrarenal malignant rhabdoid tumor (ER-MRT) (4). Recent studies indicate that malignant rhabdoid tumors (MRT) have a very poor prognosis, with a 5-year overall survival rate of approximately 15%−20%. Among these, the 5-year survival rate for MRTK is approximately 20%−25%, and for ATRT, it is approximately 32%−50% (6, 7). Due to the rarity of this disease, no unified international treatment standard has been established. Treatment typically involves surgery as the primary approach, supplemented by radiotherapy, chemotherapy, and autologous stem cell transplantation, but the efficacy is limited and requires individualized adjustments (8).

In global neonatal oncology practice, the International Society of Paediatric Oncology (SIOP) promotes a multidisciplinary collaborative model, emphasizing the integration of comprehensive treatment and nursing care to optimize treatment outcomes and care quality for pediatric cancer patients (9). These standards include precise perioperative management, psychosocial support, and family-centered humanistic care, with a particular emphasis in neonatal oncology nursing on the physiological vulnerability of preterm infants, complication prevention, and long-term prognosis management (10). However, ER-MRT, as an extremely rare and highly aggressive pediatric tumor, has limited literature reports, with clinical nursing experience in preterm infants being almost nonexistent. Existing studies primarily focus on the pathological features, molecular mechanisms, and treatment strategies of MRT, with few exploring nursing practices for ER-MRT in preterm infants. This results in a lack of targeted clinical nursing guidelines, particularly regarding how to balance the fragile physiological state of preterm infants with the complex conditions caused by rapid tumor progression, how to effectively manage perioperative complication risks, and how to provide psychosocial support for families, leading to significant knowledge gaps.

Nursing care for patients with ER-MRT faces multiple challenges, including: (a) low birth weight, immature organ development, and compromised immune function in preterm infants, which increase the difficulty of treatment and nursing; (b) rapid tumor progression causing swift changes in condition, necessitating heightened vigilance for complications; (c) complex issues such as perioperative infections, malnutrition, and pain management; and (d) the immense psychological burden on families due to the rarity of the disease and poor prognosis, urgently requiring psychosocial support. Compared with the well-established psychosocial support systems in neonatal oncology nursing in Western countries, such as the standardized psychological assessments (e.g., Psychosocial Assessment Tool) and interventions (e.g., cognitive behavioral therapy) recommended by the Children's Oncology Group, China faces significant gaps in implementing standardized psychosocial support tools and structured long-term follow-up mechanisms, particularly due to limited resources and underdeveloped community-based care (11, 12).

In July 2023, our department admitted a preterm infant diagnosed with ER-MRT. Through multidisciplinary collaboration, a precise surgical plan and comprehensive nursing strategies were developed, including preoperative preparation, intraoperative coordination, postoperative recovery, and family support. The patient's condition improved 15 days post-surgery, leading to a successful discharge. However, tumor recurrence was detected during a follow-up at 3 months of age, and the patient tragically passed away on February 17, 2024, due to intracranial metastasis and secondary brain herniation, with a total survival period of 214 days.

This case study presents a comprehensive account of the multidisciplinary management of a preterm infant diagnosed with the rare and aggressive ER-MRT. We detail the complete clinical course—from initial presentation and diagnosis, including key pathological and genetic findings, through to surgical treatment and the final outcome. Within this essential medical context, our primary focus is to systematically describe and analyze the targeted perioperative nursing strategies and humanistic care measures that were implemented. The objectives of this report are to: (a) explore the unique nursing challenges posed by this vulnerable patient population; (b) highlight the critical role of the multidisciplinary team in optimizing both treatment and care; and (c) evaluate the impact of integrated psychosocial support for the family facing a devastating prognosis. This article seeks to provide practical guidance for the comprehensive nursing care of patients with ER-MRT and underscores the complexity and significance of medical treatment and humanistic care for this rare disease. The case is reported as follows:

## 2 Case presentation

### 2.1 General information

The patient, a 4-day-old female infant, was born at 35^+6^ weeks gestation via cesarean section on July 23, 2023, due to “fetal structural abnormalities and intrauterine growth restriction”. She was the second pregnancy (G_2_P_1_) of her mother, with a birth weight of 2,250 g and Apgar scores of 9, 10, and 10 at 1, 5, and 10 min, respectively. The mother had a history of influenza A infection and fungal vaginitis during pregnancy (vaginal secretion culture negative for *Group B Streptococcus*), as well as a prior *SARS-CoV-2* infection with fever and rash. Additionally, the mother was diagnosed with gestational hypertension and elevated blood glucose (managed through diet and exercise) and had one previous spontaneous miscarriage (fetal demise). Both parents were healthy, not consanguineous, and had no family history of genetic disorders or tumor-related diseases. Prenatal ultrasound on July 15 revealed bilateral soft tissue masses in the fetal neck and shoulder regions (right side: 6.1 × 2.0 cm; left side: 6.0 × 2.3 cm), with color Doppler flow imaging (CDFI) showing abundant blood flow signals. A follow-up ultrasound on July 20 confirmed a solid mass in the left neck-shoulder region. Upon birth, the infant was immediately transferred to the Neonatology Department, where she received protective isolation, sterile petrolatum gauze coverage of the mass, anti-infective treatment with ampicillin and cefotaxime, and acetaminophen for pain relief. On July 27, she was admitted to the Neonatal Intensive Care Unit (NICU) of our hospital.

**Figure 1:**
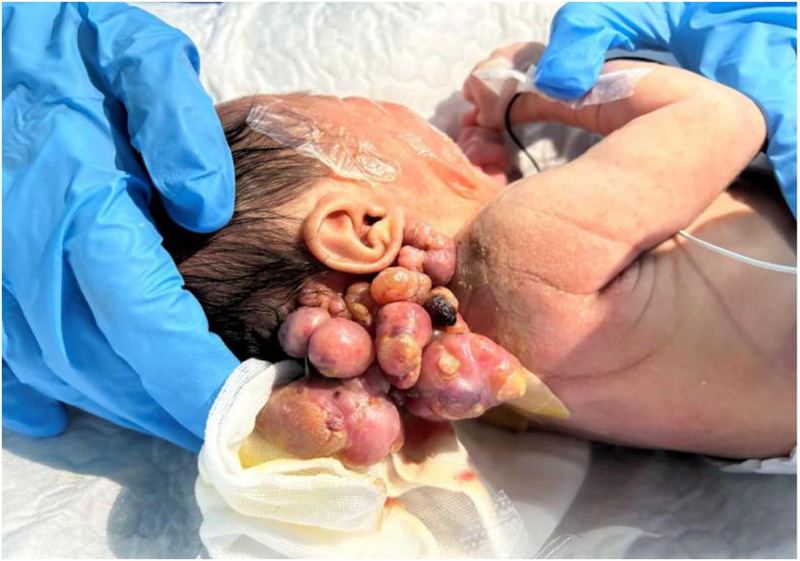
Right temporo-occipital-cervical mass.

**Figure 2:**
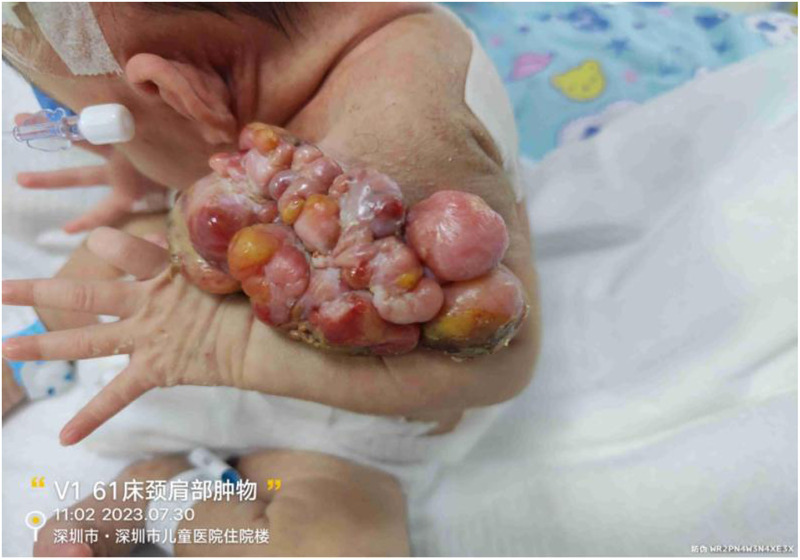
Left shoulder mass.

**Figure 3:**
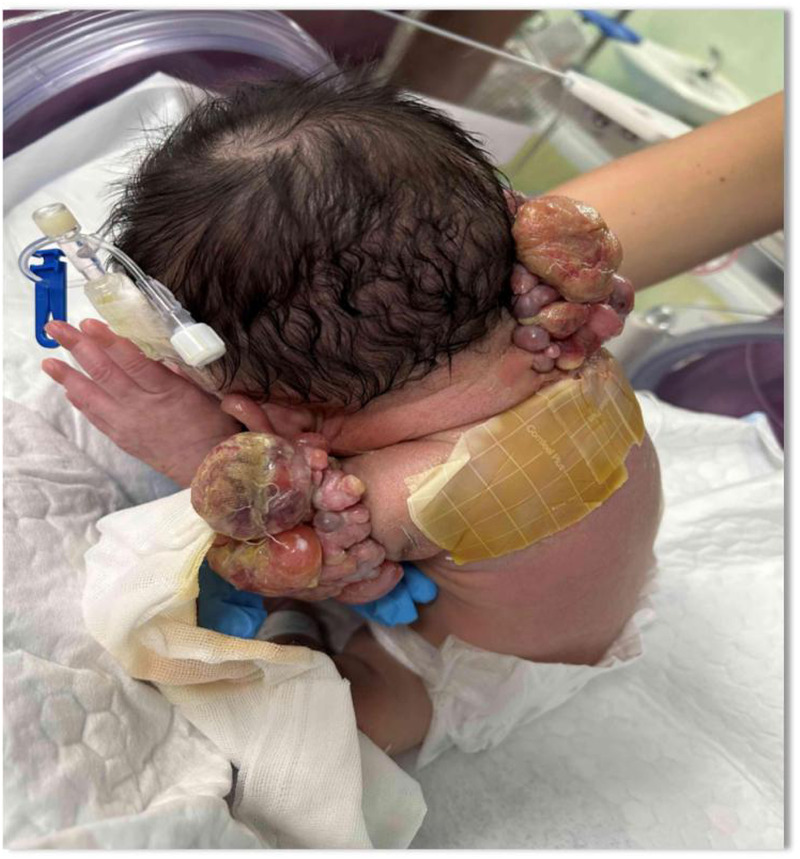
Right temporo-occipital-cervical and left shoulder masses.

On admission, physical examination revealed fair mental responsiveness, mildly rapid breathing, and warm extremities. Multiple flesh-colored masses of varying sizes were observed on the right occipito-cervical region and left shoulder, arranged in a beaded pattern, protruding from the surface with unclear boundaries. The right occipito-cervical mass measured approximately 10.5 × 5.5 × 6.0 cm, and the left shoulder mass measured about 8.0 × 5.0 × 6.0 cm. Some areas showed ulceration with oozing blood, a dark red appearance, and a foul odor (Figures 1–3). On August 1, the infant underwent surgical resection of the left shoulder mass, resection of the right occipital mass, random flap reconstruction, and left axillary lymph node dissection under general anesthesia.

### 2.2 Diagnosis, treatment, and outcome

Upon admission, the infant was immediately placed under continuous cardiac monitoring, and comprehensive laboratory and imaging examinations were conducted. A multidisciplinary team (MDT) involving general surgery, neurosurgery, hematology, and dermatology was consulted. The general surgery team recommended an initial tumor biopsy, which was performed bedside on the shoulder mass that afternoon. The dermatology team provided guidance on managing the ulcerated skin: disinfection with 0.5% povidone-iodine followed by topical application of mupirocin ointment (twice daily). The following day, through an expedited green channel, CT scans and MRI of the neck, shoulder, and brain were completed, revealing multiple nodular soft tissue lesions in the right occipito-cervical region, left shoulder, axilla, and upper arm subcutaneous tissue, suggestive of vascular malformations with possible hemorrhage. Laboratory tests showed the following: lactate 2.6 mmol/L, alkaline phosphatase 136 U/L, gamma-glutamyl transferase 65 U/L, and lactate dehydrogenase 207 U/L. Tumor markers included alpha-fetoprotein (AFP) at 50,380 ng/ml (reference range: 0.0–2,000 ng/ml), carcinoembryonic antigen (CEA) at 1.5 ng/ml (reference range: 8.1–62 ng/ml), and neuron-specific enolase (NSE) at 29.6 ng/ml (reference range: 21–39.2 ng/ml). Inflammatory markers were elevated: white blood cell count 17.39 × 10^9^/L (reference range: 9.0–30.0 × 10^9^/L), C-reactive protein (CRP) 49.2 mg/L (reference range: 0.0–8.0 mg/L), procalcitonin 0.37 ng/ml (reference range: 0.00–0.15 ng/ml), and interleukin-6 (IL-6) 116 pg/ml (reference range: 0.00–6.00 pg/ml). The infant received a 7-day course of anti-infective therapy with penicillin and ceftazidime. Given the mother' s history of fungal vaginitis during pregnancy and the confirmation of *Candida albicans* infection in the infant's mass secretions via culture, a 5-day course of antifungal treatment with fluconazole injection was administered.

**Figure 4:**
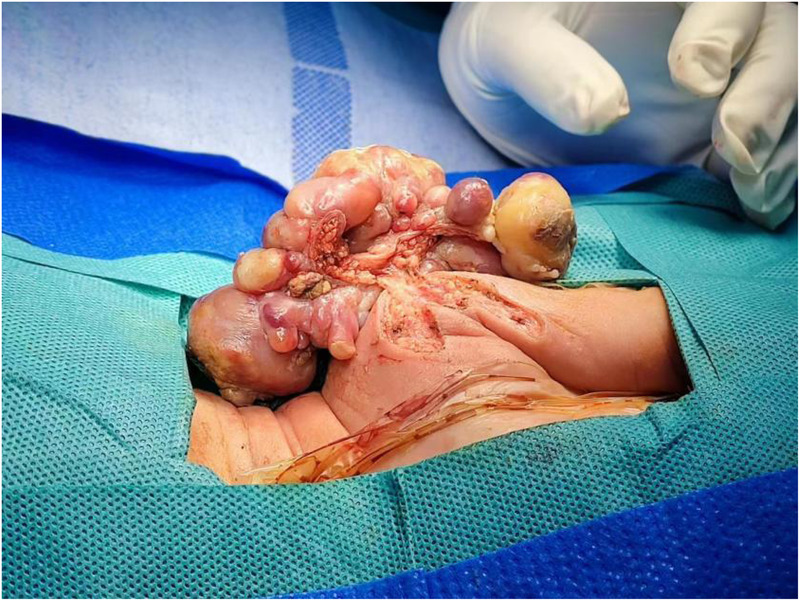
Intraoperative tumor findings.

**Figure 5:**
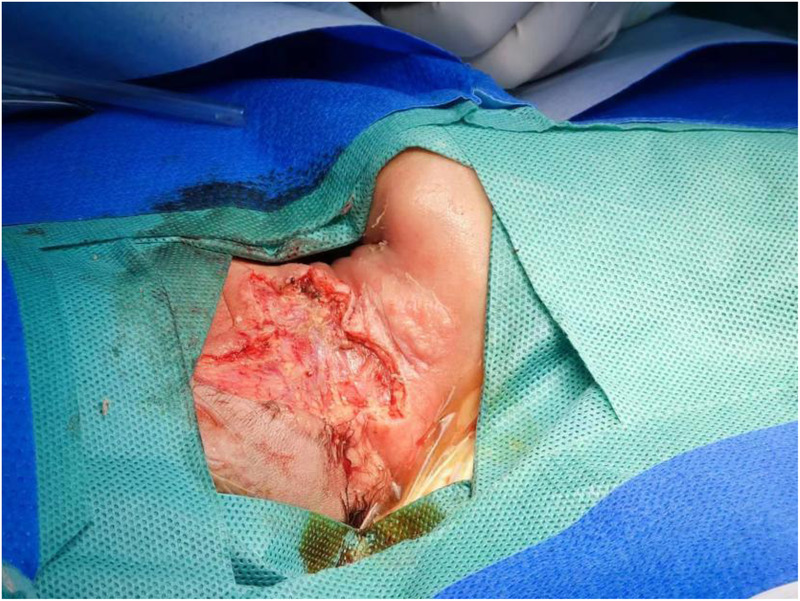
Post-tumor resection.

**Figure 6:**
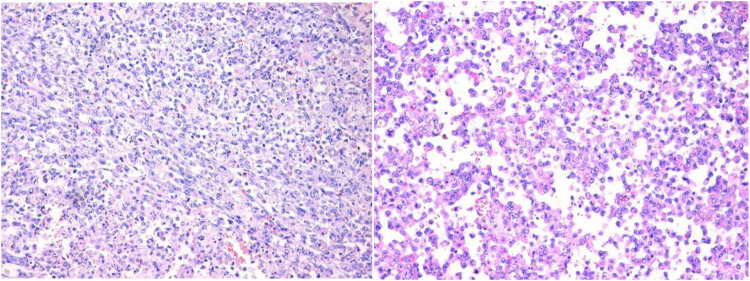
Tumor cell pathology examination. Annotation: The tumor cells are diffusely and sheet-like arranged, composed of round and oval cells. The tumor cell nuclei are large, with multinucleated tumor cells observed. The nuclei exhibit a vacuolated appearance with prominent nucleoli. The cytoplasm is eosinophilic or basophilic, and pathological mitotic figures are readily observed.

Intraoperative exploration revealed that the tumor did not invade the muscle or deep fascia (Figures 4, 5), but the left axillary lymph nodes were enlarged. Pathological examination showed that the tumors in the left shoulder and right occipito-cervical regions were *INI-1*-deficient (Figure 6). Combined with morphological and immunohistochemical features, a diagnosis of ER-MRT was confirmed. The left axillary lymph nodes exhibited reactive hyperplasia, with no evidence of tumor cell infiltration. Postoperatively, the infant received mechanical ventilation for 2 days, with continuous monitoring of heart rate, respiratory rate, oxygen saturation, and invasive arterial blood pressure. Dopamine and dobutamine were administered to improve circulation, while midazolam and fentanyl were used for sedation and analgesia. The infant' s gastrointestinal function remained intact, allowing enteral feeding to commence 24 h post-surgery.

Fourteen days post-surgery, the sutures were removed, and the infant was discharged uneventfully on August 15, 2023. At discharge, the infant was exclusively breastfed orally, receiving 40 ml every 3 h, with a weight increase to 2,690 g.On July 28, 2023, blood was drawn for family-enhanced whole-exome sequencing to screen for genetic disorders. The report, available on August 15, 2023, revealed a heterozygous deletion in the 22q11.23 region (chr22:24,085,986–24,709,434) involving the *SMARCB1* gene. This finding supported the diagnosis of ER-MRT and suggested the potential presence of rhabdoid tumor predisposition syndrome (RTPS). Genetic counseling and regular follow-up were recommended.

At the 3-month follow-up, a millet-sized, flesh-colored mass was observed at the surgical scar on the neck. Imaging and immunological examinations indicated tumor recurrence. The family, having been informed of the potential poor prognosis during earlier discussions, declined further surgery or chemotherapy. On February 15, 2024, the infant was readmitted due to intracranial metastasis of ER-MRT. The family refused invasive treatments, and the infant was declared clinically deceased on February 17, 2024, after a survival of 214 days.

## 3 Nursing management strategies

### 3.1 Prevention and control of fungal infection

The infant, a preterm neonate with compromised immunity, had a maternal history of fungal vaginitis during pregnancy. Culture of the secretions from the neck and shoulder mass confirmed *Candida albicans* infection. In neonates, particularly preterm infants, untreated fungal infections can invade the central nervous system, leading to meningitis, or progress to fungal sepsis, both associated with high transmission and mortality rates (13, 14). Additionally, fungal infections are prone to contact transmission, posing a risk of nosocomial spread (15). To mitigate this, contact isolation measures were implemented: the infant was placed in a single isolation room with dedicated nursing care, and strict disinfection and isolation protocols were enforced. Healthcare providers adhered to standard precautions, including wearing protective clothing and gloves, performing rigorous hand hygiene before and after contact, and washing hands with running water after glove removal. The infant' s belongings were designated for exclusive use and disinfected immediately after contact to prevent fungal transmission.

For anti-infective treatment, the infant received penicillin injection at 100,000 U/kg every 12 h and ceftazidime injection at 50 mg/kg every 12 h. Concurrently, antifungal therapy with fluconazole injection was administered, starting with a loading dose of 12 mg/kg, followed by 6 mg/kg daily. Following dermatology consultation, the affected skin was disinfected daily with 0.5% povidone-iodine, followed by topical application of mupirocin ointment twice daily to prevent local infection. Continuous cardiac monitoring was maintained, with close observation of vital signs, including monitoring for fever, increased heart or respiratory rates, apnea, or decreased oxygen saturation, to detect systemic infection symptoms and prevent septic shock.

### 3.2 Bedside biopsy specimen collection

To assist the surgeon in obtaining a bedside biopsy specimen, the procedure was performed under continuous sedation and analgesia, adhering strictly to aseptic techniques to prevent infection spread. One nurse positioned the infant in the right lateral position, while another adjusted the incubator to radiant warmer mode, using skin temperature control to maintain 37°C and prevent heat loss due to convection. The mass was cleansed with saline preheated to 37°C, and the general surgeon disinfected the shoulder area with 0.5% povidone-iodine before excising a specimen measuring approximately 2 × 1 × 1 cm^3^. The specimen was immediately fixed in formalin and sent for pathological examination. The incision site exhibited approximately 0.5 ml of oozing blood, after which the area was disinfected again and covered with sterile gauze.

### 3.3 Multidisciplinary collaboration to protect tumor integrity

A multidisciplinary team, consisting of three neonatology nurses, two general surgeons, one dermatologist, and one wound and ostomy specialist nurse, collaborated to develop a skin protection plan. Two early studies suggest that topical petrolatum ointment may increase the risk of candidemia and *coagulase-negative staphylococcal* infections in extremely low birth weight infants (16, 17). Therefore, in neonatal oncology care, such occlusive dressings should be used cautiously to reduce infection risk, and the use of petrolatum gauze coverage should be discontinued. Plan A involved wrapping the tumor with a Silo bag, a technique commonly used for staged repair of giant omphalocele or gastroschisis in neonates (18). Following multidisciplinary discussion, the infant was positioned in a three-tier prone position (head on the highest tier, chest on the second tier, and lower limbs on the third tier), with Silo bags applied to wrap the neck and shoulder tumors separately. However, after 2 h in the prone position, the tumor compressed the neck, shoulder, and surrounding tissues. Despite continuous sedation with midazolam, the infant exhibited restlessness, with heart rate fluctuating between 175 and 190 beats/min, respiratory rate at approximately 60 breaths/min, and oxygen saturation varying between 84% and 92%. Consequently, this plan was aborted.

Plan B was then adopted, involving alternating left and right lateral positions, with the use of a fluid pad, sterile maternity pads, and foam dressings for protection. The fluid pad was placed beneath the maternity pad, allowing positional adjustments through its malleability. When in the left lateral position, the infant' s left upper limb was abducted at 30–45 degrees with the arm naturally internally rotated to prevent pressure on the tumor site. The Neonatal Skin Risk Assessment Scale (NSRAS) was used daily to evaluate the risk of pressure injuries, yielding a score of 17, indicating a high risk of pressure injury (19). Sterile maternity pads were placed between the bed sheet and the infant, and sterile foam dressings were applied to the shoulder and neck tumor sites to minimize pressure and friction, preventing epidermal breakdown. The skin around the tumor was continuously monitored for changes in color, as well as for signs of bleeding, exudation, or purulent discharge. No new ulcerations developed on the tumor prior to surgery in this infant.

### 3.4 Perioperative temperature management

In neonates, the thermoregulatory center is immature, with a large body surface area, thin subcutaneous fat, and reliance on brown adipose tissue for heat production, which is primarily distributed in the neck, supraclavicular region, mediastinum, paraspinal areas, and around the kidneys (20, 21). The infant in this case had a large tumor located in the neck and shoulder region, increasing heat loss due to intraoperative skin exposure, compounded by the effects of anesthetic drugs and other factors (22, 23), thus posing a risk of perioperative hypothermia.

A collaborative strategy to prevent hypothermia was developed with the anesthesiologist, surgeon, and operating room nurses, as follows: (a) During transport, an incubator with a backup battery was used to ensure continuous power supply, maintaining the infant' s normal body temperature. (b) Thirty minutes prior to surgery, the operating room temperature was adjusted to 26–30°C to minimize heat loss through radiation and convection. (c) Core body temperature was dynamically monitored perioperatively, with a rectal probe inserted 2–3 cm, keeping the infant' s rectal temperature between 36.2 and 36.9°C. (d) Intraoperative skin disinfectants and saline irrigation fluids were preheated to 37°C, and intravenous fluids were warmed to 36–37°C using an infusion warmer before administration. (e) After returning to the ward post-surgery, the humidified gas temperature in the ventilator was maintained at 36.5–37.3°C. All procedures were performed in a consolidated manner to minimize frequent opening of the incubator door, reducing heat loss, and a “nest” was used for additional warmth.

### 3.5 Stepwise pain management

Based on the 2023 Chinese Evidence-Based Guidelines for Neonatal Pain Management (24), a stepwise pain management approach was adopted, integrating pharmacological and non-pharmacological interventions to develop a personalized plan. Postoperatively, the infant returned to the ward with an endotracheal tube in place and received positive pressure ventilation via a resuscitation bag, as anesthesia had not yet worn off. Per medical orders, midazolam injection at 0.3 µg/(kg·min) and fentanyl injection at 1 µg/(kg·h) were administered via continuous intravenous infusion for sedation and analgesia until invasive mechanical ventilation was discontinued. Pain was assessed every 6 h using the Neonatal Postoperative Pain Scale (CRIES) and the Neonatal Pain, Agitation, and Sedation Scale (N-PASS). The CRIES score was 3, the N-PASS pain score ranged from 3 to 4, and the sedation score was −2, indicating mild-to-moderate pain and a mild sedation state.

The attending physician dynamically adjusted the medication doses based on these scores to ensure effective sedation and analgesia, alleviating the infant' s pain while avoiding oversedation. Daily assessments by the medical and nursing team evaluated criteria for extubation to minimize persistent pain. After 2 days of invasive mechanical ventilation, the infant was transitioned to 25% oxygen supplementation within the incubator. Non-pharmacological interventions were then implemented, including playing pre-recorded gentle maternal speech or lullabies, as well as soothing music, with the sound intensity controlled at 35 dB and delivered inside the incubator to relieve pain. Prior to procedures that could cause acute pain, such as wound dressing changes or blood glucose monitoring, non-nutritive sucking was also provided to further mitigate discomfort.

### 3.6 Postoperative wound care

Following the extensive tumor resection, the infant' s wound skin tension was closely monitored to prevent surgical incision dehiscence. The wound was inspected during each nursing shift, with the head nurse assisting in evaluating the healing progress and ensuring proper postoperative wound disinfection. After disinfecting with 0.5% povidone-iodine, the wound and surrounding skin were wiped with saline to reduce iodine absorption and mitigate potential effects on the preterm infant' s thyroid function (25, 26). Within the first 48 h post-surgery, the wound exhibited significant oozing of blood and exudate. Sterile gauze was weighed to accurately measure the exudate volume. On the first postoperative day, the wound exuded approximately 9 ml of pale yellow fluid mixed with blood; on the second day, the exudate consisted of about 5 ml of pale yellow and light pink fluid.

## 4 Humanistic care for the Neonate's family

### 4.1 Psychological support and communication during hospitalization

Families of neonates typically experience great anticipation and joy at the arrival of a new life. However, in this case, the mother underwent regular prenatal checkups, and a late-pregnancy ultrasound revealed a large mass in the fetal neck and shoulder region. Postnatally, the pathology confirmed the diagnosis of ER-MRT. Primary MRT of the neck and shoulder in a fetus is extremely rare and carries a very poor prognosis, making it emotionally challenging for the parents to accept. The parents lacked knowledge about the disease, exhibited significant anxiety, and were eager to understand the treatment, care, and prognosis. The Hamilton Anxiety Rating Scale (HARS) was used to assess the psychological state of the infant' s parents, revealing a score of 31 for the mother (indicating severe anxiety) and 16 for the father (indicating moderate anxiety) (27). A psychologist from the hospital' s social work department was invited to provide psychological counseling, aiming for early identification and prevention of posttraumatic stress disorder (PTSD) in the mother. Research indicates (28, 29) that PTSD significantly impairs the physical and mental health of parents of children with malignant tumors, potentially hindering their ability to make accurate treatment decisions and diminishing their capacity to provide daily care and emotional support to their children. During communication, we observed that the mother experienced immense psychological stress, with her initial joy over the new life turning into fear of tumor recurrence and death. This distress was exacerbated by insufficient family and social support, disruptions to daily life, and increased financial burdens.

To improve doctor-patient communication, we established a WeChat group comprising the attending physician, primary care physician, head nurse, and the infant' s family, facilitating timely sharing of multidisciplinary consultation outcomes and treatment-care plans. Post-surgery, the parents were invited to the NICU for bedside visits, allowing us to understand their needs, provide emotional reassurance, and patiently listen to and address their primary concerns. The mother was guided on proper lactation techniques and encouraged to continue breastfeeding, with daily delivery of breast milk to the hospital during the infant' s admission. Prior to discharge, targeted guidance was provided, focusing on wound observation and care techniques. The parents' home caregiving capabilities were assessed using the Wenjuanxing platform (https://www.wjx.cn/), ensuring they possessed essential nursing skills.

### 4.2 Post-discharge home visits and support

The infant's father had come to understand and accept the reality of the child' s rare malignant tumor and poor prognosis. Given the infant' s preterm status and underdeveloped liver and kidney function, which posed a high risk for systemic chemotherapy, the father declined this treatment. Although the mother agreed with the father' s decision, she struggled emotionally, exhibiting severe anxiety and low mood. The attending physician and head nurse conducted home visits on the 7th day, 15th day, 1st month, and 2nd month post-discharge, providing guidance on home environment setup, warmth maintenance, feeding precautions, and complication monitoring, while also tracking the infant' s growth, development, and disease progression, and offering ongoing psychological support to the family. Regular follow-ups with the general surgery and hematology departments were scheduled post-discharge. At the 1-month follow-up, tumor marker tests revealed an AFP level of 1,687.8 ng/ml (reference range: 0.0–2,000 ng/ml) and a NSE level of 33.8 ng/ml (reference range: 21–39.2 ng/ml). During the 2-month home visit, the infant' s weight was 4,900 g, length was 56 cm, and head circumference was 40 cm, indicating good growth and development. The mother's anxiety score had decreased from 31 at admission to 17, and she proactively inquired about future health concerns, such as the appropriate timing for another pregnancy and the medical consultations and tests required for both parents. Over time, the parents gradually came to accept the unfavorable outcome calmly, and with their consent, subsequent home visits were discontinued.

Perioperative events, outcomes, and comprehensive nursing interventions are summarized in Table 1.

**Table 1:** Summary of perioperative events, outcomes, and comprehensive nursing intervention.

Phase	Time point	Nursing interventions	Clinical outcomes	Parental psychological status
Preoperative	Admission to Biopsy Period	• Fungal infection control: Single-room isolation, penicillin (100,000 U/kg, q12h) + ceftazidime (50 mg/kg, q12h) + fluconazole (loading dose 12 mg/kg, then 6 mg/kg/day • Skin care: Application of mupirocin (twice daily) after disinfection with 0.5% povidone-iodine • Multidisciplinary consultation (general surgery, neurosurgery, dermatology, hematology) to develop a personalized plan	• *Candida albicans* infection controlled, no nosocomial transmission • Tumor integrity maintained, no new lesions • Biopsy successful, specimen sent for examination, stable body temperature, no hypothermia • Alpha-fetoprotein 50,380 ng/ml, carcinoembryonic antigen 1.5 ng/ml	• Mother's HARS score: 31 (severe anxiety) • Father's HARS score: 16 (moderate anxiety)
Postoperative	Postoperative Days 1–2	• Pain management: Midazolam (0.3 μg/kg·min) + fentanyl (1 μg/kg·h) intravenous infusion, CRIES/N-PASS scoring (every 6 h) • Skin care: 0.5% povidone-iodine disinfection followed by saline wipe, covered with sterile gauze • Wound exudate monitoring, weighing gauze	• CRIES score 3, N-PASS score 3–4 (mild to moderate pain) • Wound exudate: 9 ml on day 1 (yellowish bloody), 5 ml on day 2 (yellowish/pink), no infection • Stable body temperature, no wound infection	• Social worker psychological counseling, mother's anxiety began to ease
Postoperative Recovery	During Hospitalization	Oral feeding: Initiated 24 h post-operation, breast milk feeding 40 ml every 3 h before discharge Growth and development monitoring: Weight, length Psychological support: NICU visitation, patient-provider communication group, breastfeeding guidance Infection markers monitoring: White blood cell count, C-reactive protein, procalcitonin, interleukin-6	• Weight increased to 2,690 g (19.6% growth) • Infection markers returned to normal, good gastrointestinal function • No hypothermia or new infections	• Risk of PTSD possibly reduced
Post-Discharge Follow-Up	1–2 Months Post-Discharge	• Family visits (days 7, 15, 1 month, 2 months): Guidance on feeding, wound care, and psychological support • Regular follow-up of tumor markers (alpha-fetoprotein, neuron-specific enolase) • Psychological support: Ongoing counseling to alleviate anxiety	• Alpha-fetoprotein 1,687.8 ng/ml (normal), neuron-specific enolase 33.8 ng/ml (normal) • At 2 months of age: Weight 4,900 g, length 56 cm, head circumference 40 cm • Disease progression, survival period 214 days (deceased on February 17, 2024)	• Mother's HARS score: 17, parents gradually accepted the poor prognosis • Proactively inquired about subsequent health issues (e.g., future reproduction)

## 5 Summary

Malignant rhabdoid tumor (MRT) is a rare pediatric tumor characterized by high invasiveness, rapid progression, and a typically poor prognosis (30). In this case of ER-MRT, a multidisciplinary team collaborated with case discussions to develop targeted strategies for fungal infection control, tumor skin protection, prevention of perioperative hypothermia, and refined pain management. The postoperative physiological recovery of the infant was significant. During hospitalization, infection-related indicators (white blood cell count, C-reactive protein, procalcitonin, and interleukin-6) all returned to normal ranges, and body temperature remained stable. Gastrointestinal function was robust (oral feeding began 24 h post-surgery, with 40 ml of breast milk fed orally every 3 h at discharge). Nutritional status improved markedly (body weight at discharge was 2,690 g, a 19.6% increase from admission). These findings suggest that comprehensive nursing strategies may have effectively promoted the infant's short-term recovery, particularly in preventing perioperative complications and supporting physiological functions. However, the infant's recovery may also be influenced by medical treatments and individual factors, necessitating further research to clarify the independent contribution of nursing interventions.

The incidence of malignant rhabdomyosarcoma in newborns is extremely low, and domestic research on the psychological status of parents of children with this disease is limited. Compared to the well-established psychosocial support systems for pediatric oncology in Europe and North America, such as the multidisciplinary team collaboration and standardized psychosocial assessment and intervention measures recommended by the SIOP and the Children's Oncology Group (COG) (31, 32), China urgently needs improvement in humanistic care and systematic psychological support for families of children with malignant tumors, including infants. Domestic studies indicate a scarcity of psychological support resources and a lack of standardized assessment and intervention tools for cognitive impairment and psychological needs (33, 34).

In this case, while treating the affected child, family psychological care was integrated throughout the process, encouraging parental involvement in medical decision-making, promptly identifying the mother's psychological stress, and engaging a professional counselor for intervention. The mother's anxiety score (HARS) decreased from 31 at admission to 17, suggesting that psychological support may help alleviate anxiety and reduce the risk of PTSD. However, relying solely on the HARS scale cannot comprehensively assess PTSD, and future studies should employ specialized diagnostic tools for validation. Post-discharge, continuous care was provided through home visits, and parental feedback indicated that the support from the care team was associated with reduced psychological stress and improved quality of life. These interventions align with the effects of psychological support reported internationally, suggesting that multidisciplinary collaboration and family-centered care have universal applicability worldwide. Nevertheless, China still faces gaps in standardized psychological support tools, long-term follow-up mechanisms, and the integration of care resources for rare diseases. Particularly in resource-limited regions, delayed diagnosis and inadequate psychological support remain global challenges (33).

This case suggests that comprehensive nursing strategies combined with humanistic care may contribute to improving treatment outcomes and family support systems for neonates with malignant tumors, providing a practical reference for the nursing care of similar cases. Future research could further explore long-term prognostic indicators for preterm infants with ER-MRT (e.g., 1-year survival rate, neurodevelopmental outcomes) and the effectiveness of standardized psychological support protocols. Additionally, it is recommended that healthcare providers promote multidisciplinary collaboration in clinical practice, develop psychological intervention toolkits tailored for families of neonates with malignant tumors, and establish regional neonatal tumor care databases to optimize care quality and data support. These measures will help address knowledge gaps in neonatal tumor care and provide clearer practical guidance for healthcare professionals.

## 6 Limitations

This study, being a report on a rare disease, has certain limitations. First, as a single-case study, the generalizability of the results may be limited. Second, the infant was preterm and had a concurrent fungal infection, which may not fully represent the clinical characteristics of other MRT or neonatal tumor patients. Third, the follow-up period was short (2 months), lacking long-term prognostic data (the infant's survival was only 214 days), such as 1-year survival rates or neurodevelopmental outcomes. Fourth, parental psychological assessment relied solely on the HARS scale, without using PTSD-specific diagnostic tools, limiting the comprehensive evaluation of PTSD risk. Additionally, the association between nursing interventions and reduced psychological stress was based on observational data, lacking a control group to establish causality. Future studies should include larger cohorts, extend follow-up duration, and employ standardized psychological assessment tools to enhance the reliability and applicability of the findings.

## Data Availability

The original contributions presented in the study are included in the article/Supplementary Material, further inquiries can be directed to the corresponding author/s.
